# Novel Soft Dosage Forms for Paediatric Applications: Can We 3D-Print Them or Not?

**DOI:** 10.3390/gels11030187

**Published:** 2025-03-08

**Authors:** Antoni Białek, Julia Krysztofiak, Aleksandra Hozakowska, Zuzanna Wojszel, Tomasz Osmałek, Monika Wojtyłko, Anna Froelich

**Affiliations:** 1 Student’s Research Group of Pharmaceutical Technology, The Student Scientific Society of Poznan University of Medical Sciences, 5 Rokietnicka Street, 60-806 Poznań, Poland; antoni.s.bialek@gmail.com (A.B.); julia.krysztofiak@icloud.com (J.K.); ahozakowska@gmail.com (A.H.); zuzanna.wojszel@gmail.com (Z.W.); 2 Chair and Department of Pharmaceutical Technology, Poznan University of Medical Sciences, 3 Rokietnicka Street, 60-806 Poznań, Poland; tosmalek@ump.edu.pl; 3 Chair and Department of Pharmaceutical Technology, 3D Printing Division, Poznan University of Medical Sciences, 3 Rokietnicka Street, 60-806 Poznań, Poland; 4 Doctoral School, Poznan University of Medical Sciences, 70 Bukowska Street, 60-812 Poznań, Poland

**Keywords:** soft dosage forms, gummies, gels, chocolate, semi-solid extrusion, gel printing, paediatric formulations, 3D-printing

## Abstract

Over the past years, numerous novel dosage forms, including gels, have been investigated for paediatric treatment due to the need to provide flexible dose adjustment possibilities, as well as a patient-friendly approach to drug delivery. Simultaneously, 3D printing technology is continuously advancing and gaining interest as a tool for personalised formulation development. Multiple additive manufacturing methods, including the semi-solid extrusion, especially used in gel printing, provide flexibility regarding the dose of active ingredients and the adjustment of the design of soft dosage forms. 3D printing techniques can be considered as a possible answer to the demand for medicines tailored to small patients’ needs. This review intends to present an overview of the current possibilities, comparing gel-like and non-gel-formulated dosage forms and crucial aspects of developing those cutting-edge dosage forms by 3D printing. This paper discusses soft formulations such as chewing gums, which still require extensive evaluation, and explores the question of the three-dimensional printing process. Furthermore, it highlights soft dosage forms, such as gel-based gummies and hydrogels, for which 3D fabrication has been intensively studied in previous years. However, the research still needs to advance.

## 1. Introduction

Developing drugs suitable for the paediatric population maintains its status as a vital challenge for regulatory stakeholders, the pharmaceutical industry, science, politics and patients. It is significant internationally since every day, millions of children are seen by healthcare professionals due to potentially fatal illnesses [1]. The need to ensure adherence by providing acceptable medicines and accessible age-appropriate drug delivery products, is growing [2].

Nowadays, healthcare professionals or caregivers face daily challenges, due to numerous non-child-friendly medicines. This often results in the adaptation of medicines intended for adults, by pharmaceutical compounding and manipulation [3]. The off-label use of drugs (outside the terms of the marketing authorisation) may lead to an increased risk of side effects [4]. Additionally, various studies have emphasized the challenge of obtaining an accurate dose from tablet manipulation [5]. Consequently, these practices cannot be regarded as an equivalent for commercially available drugs, produced according to Good Manufacturing Practice [6]. Nonetheless, involving the dose adaptation by pharmacists, nurses or parents remains the most commonly used solution to the lack of therapeutics for children [7]. These practices pose significant risks and may cause harm, including a lack of therapeutic effectiveness and toxicity [8].

Currently, the children population, that is people under 18 years old, is 2,415,319,658 [9], with around 654,028,000 under 5 years old [10]. In this paediatric population, we can distinguish several groups based on their age [11] (in completed days, months or years). Although there is some variation in how these age groups are categorized, the International Congress on Harmonization (ICH) offers a commonly used classification. According to the ICH E11 guidance, the paediatric populations can be divided into: preterm newborn infants, term newborn infants (0 to 27 days), infants and toddlers (28 days to 23 months), children (2 to 11 years) and adolescents (dependent on region: 12 to 16–18 years). Age classification is based on overlap in developmental issues, however, it is to some degree arbitrary. Therefore, a flexible approach is essential in studies.

The issue of billions of paediatric patients still lacking access to appropriate therapy prompted stakeholders to introduce new regulatory frameworks as an incentive for the development of child-friendly medicines. Nowadays, the pharmaceutical industry must include children in the drug development process with a Paediatric Investigation Plan (EU) or Paediatric Study Plan (USA). Moreover, multiple regulations have been implemented by the United States of America, including Title V of the Food and Drug Administration Safety and Innovation Act (FDASIA) [12], which reauthorizes and amends provisions of the Best Pharmaceuticals for Children Act (BPCA) and the Paediatric Research Equity Act (PREA). Similarly, the European Union introduced the EU Paediatric Regulation: (EC) No. 1901/2006 and (EC) No. 1902/2006 [13] and the World Health Organisation launched the Make Medicines Child Size initiative [14]. Simultaneously, several initiatives, including the European Paediatric Formulation Initiative (EuPFI; 2007) [8] and the Global Accelerator for Paediatric Formulations (GAP-f) [15] focused on age-appropriate drug formulation development. Moreover, numerous organisations conducted workshops, such as the workshop on Paediatric Formulation Development: Challenges of Today and Strategies for Tomorrow [2] and the 6th APV Winter Conference Patient-Centric Drug Product Development [16], to tackle the issues of formulations tailored for paediatric patients. Across the previously mentioned workshops and initiatives, there is a recurring issue of lack of acceptability and willingness of paediatric patients to use the drugs.

Acceptability is a critical attribute to ensure paediatric patients’ adherence to drugs, according to 90% of paediatricians [2]. Commonly used formulations, such as tablets or oral liquids may be problematic due to unpleasant taste or tablet size, reducing children’s acceptability [15]. Conventional tablet use in children is also limited by the risk of choking or aspiration and the restriction of available doses [3]. In contrast, novel soft dosage forms may be an interesting solution, providing paediatric patients with an acceptable drug product. Illustratively, orodispersible dosage forms and chewable tablets are considered the dosage forms of choice for school-age children (6–11 years) and adolescents. Among these novel drug delivery approaches, the use of gels is observed [17]. Gels can be described as semisolid or elastic solid systems composed of three-dimensional lattices interpenetrated by liquid domains formed by solutions or suspensions of small inorganic particles. As gelling agents, various types of polymers can be applied, including cellulose derivatives, alginates, gums, carrageenans [18] and gelatine. Gel-based formulations have numerous advantages, such as size, shape and dose flexibility, as well as the fact that they can be easily swallowed. In addition, pharmaceutical forms, such as chewing gums, may be suitable for paediatric dosage forms, since they are easy to administer, do not require water and most children are familiar with them.

Soft novel drug delivery systems (DDSs) may be divided into gel-formulated and non-gel-formulated based on the composition of the medicine and its physicochemical properties. However, this division is to some extent arbitrary since there is no strict categorization. For the purpose of this article, various factors, including the specific composition, concentration, interaction of excipients, method of development and the original research authors’ incentive to name the DDS as a gel were considered.

There is no perfect commercially available formulation that is capable of meeting the needs of all age groups of paediatric patients [3]. However, three-dimensional printing (3DP) can be a useful tool for developing customized drug delivery for paediatrics [19]. Also referred to as additive manufacturing (AM), the 3DP technology provides vital attributes that distinguish it from other conventional manufacturing processes, including personalization, on-demand manufacturing and product complexity [20]. Three-dimensional printing offers geometrical flexibility of drugs, such as size, appearance, shape and design to address the paediatric patient desires and potentially increase compliance. Furthermore, this technique enables optimization of the dose according to the gender, age, weight, genetic profile and disease severity of the treated patient [21]. This paper discusses the development of 3D printing in paediatrics, focusing on the novel soft dosage formulation. This review intends to present an overview of current possibilities in additive manufacturing, comparing gel-like and non-gel-formulated dosage forms.

## 2. Methods of 3D Printing

**Figure 1:**
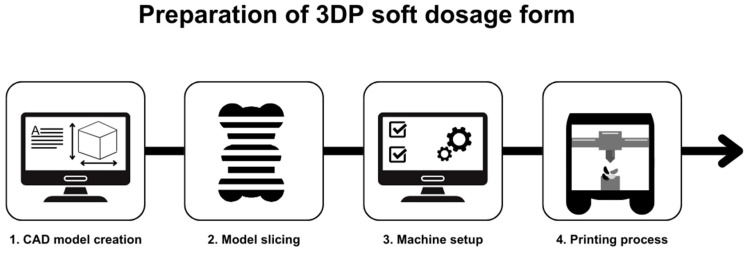
Diagram showing the steps in developing a 3D-printed paediatric drug.

Three-dimensional printing is an additive manufacturing technology, used to fabricate various structures and complex geometries from model data. This technique fabricates structures by depositing materials layer by layer onto a substrate using computer-aided design (CAD) software (Figure 1). The technology was developed by Charles Hull in 1986 [22]. However, its application has grown significantly since the 80s [23]. Three-dimensional printing is considered an umbrella term, which consists of numerous different technologies [24]; the most important ones are explained in this chapter.

### 2.1. Fused Deposition Modelling (FDM)

Fused Deposition Modelling (FDM) is a well-known hot-processing method investigated widely as a potential tool for manufacturing individual dosage forms. It can be applied successfully due to its high functionality, repeatability and exactness. Thermoplastic polymers, such as polyvinylpyrrolidone (PVP), polyvinyl alcohol (PVAl) and polylactic acid (PLA), are used to obtain an essential material of this technology—the homogeneous filament [25,26]. It is commonly produced using hot melt extrusion (HME), which can additionally improve the solubility of poorly soluble drugs [20,27,28]. The filament is molten in the heated printer block, and its subsequent layers harden and settle one on top of the other, then combine to create the final product [20,29]. Important factors affecting the definitive properties of the printing product are changes in the temperature of the process steps, the speed of execution, and the thickness of the layers [30]. Due to the requirement of this method, it is limited to the use of thermoplastic polymers only and active ingredients that are not susceptible to thermal degradation. However, the possibility of lowering the temperature in the FDM technique is being explored, which could broaden the range of materials used [26].

### 2.2. Stereolithography (SLA)

Stereolithography (SLA) is one of the first 3D-printing methods that found application in medicine, especially in dentistry [31,32]. It belongs to non-thermal techniques. The main idea of this technology is the solidification of liquid resin using photopolymerization. The UV light beam undergoes specialized control to ensure the correct light distance between the cured layers, which are formed on a moving platform. Among the photoresins used, poly(ethylene glycol) diacrylate and polycaprolactone dimethylacrylate are mentioned [33]. In addition to the lack of heat requirement, which offsets the risk of drug degradation, this method is distinguished by a short printing time, the ability to produce geometrically demanding structures, and a high-quality edge finish [34,35]. A limitation posed by the SLA technique is the need to use UV-light-curable materials, which precludes the use of some resins, as not all have such properties. The safety of using these substances for pharmacotherapeutic purposes may also be a problem [36].

### 2.3. Embedded 3D Printing (e-3DP)

Embedded 3D Printing (e-3DP) is an emerging technology which enables the printing of three-dimensional structures directly into existing material. This method is based on extruding viscoelastic ink via a deposition nozzle at a predefined path directly into the elastomeric reservoir known as the embedding phase. A nozzle moving through the solidifying reservoir creates space filled with the capping layer. After printing, the embedding phase has to be solidified via a curing method to form a monolithic part, whereas the viscoelastic ink, the embedded phase, remains fluid [37,38]. The formation of the filament is affected by matrix rheological properties and printing parameters [39]. This method is based on using two different materials, which can be successfully applied in paediatric dosage forms since children often struggle to comply with drugs due to an unpleasant taste. In this case, an active pharmaceutical ingredient (API) can act as an embedded phase while excipients with acceptable taste will be in the embedding phase. However, combining two different formulations might be challenging and hard to optimise. The limitation of e-3D printing is that the embedded object cannot retain its shape without the backing material. The resolution of e-3DP relies on the size of the needle and the particles in the medium [40].

### 2.4. Inkjet 3DP (IJP)

IJP printing was the first technique introduced into the pharmaceutical area. It is based on a liquid material jetted on [41]. It requires low-viscosity material. The process is divided into the forming droplets, ink droplet deposition and solidification stages. As liquid drops hit the surface, they solidify to form a homogeneous thin layer with fine accuracy. IJP involving a powder bed is a technique known as the drop-on-powder (DOP). In the process, a liquid binder layer is deposited precisely on a powder bed and then a powder layer is spread on top. The procedure is repeated as long as the final object is achieved. This additive technique may rely on the continuous droplet stream, where a catcher controls unwanted jetting droplets, or it may be based on a system droplets-on-demand (DOD). The DOD system is adjusted to the type of printed formulation [41]. While DOD technology uses droplets with sizes in the range from 10–50 μm and volumes in the range of 1–70 pL, continuous inkjet technology uses high-pressure pumps to obtain a continuous flow of ink through an aperture with a diameter in the 50–80 μm range [42]. Inkjet printing is a precise method in dosing and deposition, which gives a high accuracy in personalised medicine [43]. On an industrial scale, this method might be limited due to its low time efficiency. The inkjet 3D printing method is limited by the physics of the process affecting the drop frequency. Replenishment rate, the rate at which the nozzle is filled up with the ink after forming drop, cannot be adjusted [44]. Another possible limitation is the nozzle blockage from inaccurate dosing [45].

### 2.5. Semisolid Extrusion (EXT or SSE)

**Figure 2:**
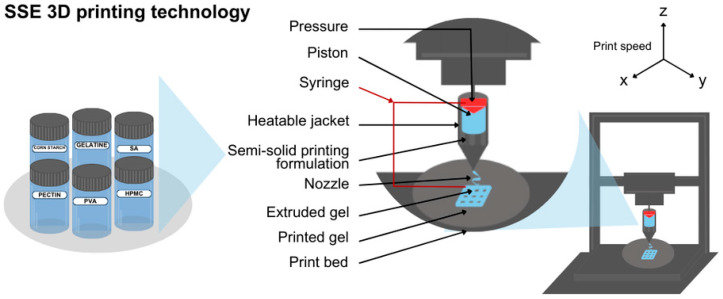
Semisolid extrusion schematic diagram.

Semisolid extrusion (SSE, Figure 2) is a technique based on extruding paste-like or gel material via a syringe-based printhead [46]. Extruding material possesses a relatively low melting point [41]. Deposited layers create the desired 3D-printed object, such as an oral dosage form for paediatric usage. SSE has advantages such as mild processing conditions including temperature, which makes this approach worth consideration in pharmaceutical manufacturing [47]. Moreover, printing can be done on the final packaging to avoid cross-contamination. The drawbacks of semisolid extrusion are low resolution and bad capacity [48]. Once extruded, the material hardens, allowing subsequent layers to be supported by those located below [49]. The limitations of the SSE technique are post-processing and high requirements for paste properties.

### 2.6. Selective Laser Sintering (SLS)

Selective laser sintering (SLS) is part of the umbrella term powder bed fusion. The mechanism of these techniques is based on melting and fusing powder particles. Specifically in SLS, the laser beam superficially sinters the thermoplastic polymers [50].

The mixture of the feedstocks, including thermoplastic polymers, in the form of homogenous powder, is located on the printer’s platform called the powder bed. The roller distributes a flat layer of the powder. During the printing process, energy from the laser beam causes heat in specific, precisely defined powder areas. Consequently, particles combine, forming the first layer of the designed object. Then, the roller redistributes the powder and repeats the sintering process layer by layer until the final result is obtained [51,52].

This method is solvent-free, which shortens the whole process, as there is no need for a drying stage. SLS’s characteristic is the ability to achieve highly controlled porosity and high resolution of the formula. Regarding security and accessibility, the important feature of this technique is the opportunity to contain commonly used pharmaceutical powders as the print’s base [50]. On the other hand, the possibility of using colourants in this method is limited, as they can interfere with the laser beam [52].

### 2.7. Digital Light Processing (DLP)

Digital light processing (DLP) is a 3DP method based on photopolymerization. The principle of operation is similar to SLA. The key is a reaction between the photosensitive resin and light irradiation. An important, unique element in DLP is a digital micromirror device. It contains a large amount of moving micro-sized mirrors that switch to on and off position. It allows whole-layer exposure to the light from the source at once [53]. The optical system makes the photons reflect and strike all the right spots. The technique requires less resin compared to SLA. These features make DLP fast and cost-efficient without compromising the quality of the print [54]. Also, DLP requires a material that is easily photo-crosslinked through light irradiation. The ink should have a relatively low viscosity. In the case of soft dosage forms, the manufacturing of complex prints might be difficult due to the risk of deformation or collapse caused by the force exerted by subsequent layers built one after another [55].

DLP provides flexibility in customizing printing parameters, such as wavelength, exposure time, UV intensity and light source [56]. However, exposure to UV light might cause photodegradation of the active compounds, affecting the API content and potentially introducing harmful products of decomposition to the formulation.

**Figure 3:**
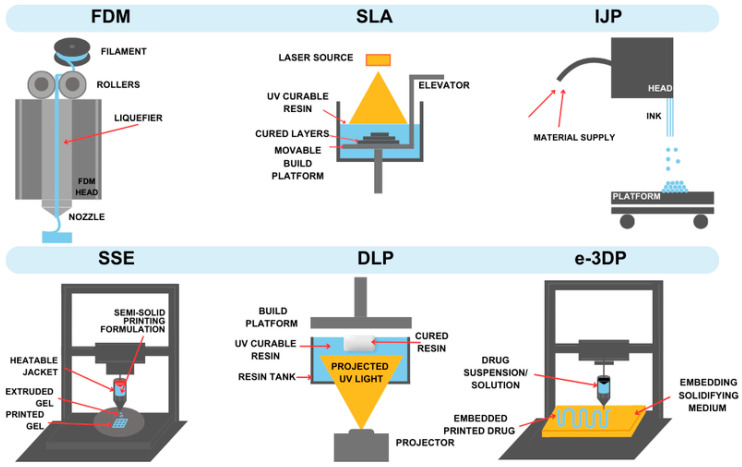
Methods of 3DP.

The schematic illustration of the most important 3D-printing techniques is presented in Figure 3, while the essential features of the methods are summarized in Table 1.

## 3. 3D-Printed Hydrogels

Gels are a promising future for individual dosage forms; for now, they are widely used in bioengineering modelling or as hydrogel dressing materials in wound healing [57]. 3D-printed hydrogels with sodium alginate, bamboo fibres and gelatine exhibit amazing healing properties and can be a useful tool in enhancing the tissue regeneration process [58]. Depending on the desired properties of the final product, gels can be obtained with the use of different polymers and different gelation mechanisms [59].

Hydrogels could be used for localised and systemic delivery, ensuring the safety and compliance of the paediatric patient. While numerous studies concentrate on transdermal and epidermal approaches to wound healing [57,60], there is a growing interest in oral dosage forms designed for paediatric patients [54]. There are a few studies where gels and hydrogels are prepared via 3DP for children. Increasing research for paediatric formulations based on gels and hydrogels is focused on oral dosing. Hydrogels can be prepared using various methods including chemical and physical crosslinking [61]. The properties of the obtained product, including porosity, swelling and release properties [62] often depend on the cross-linking density [63] and polymer selection, whereas the 3DP fabrication offers control over geometries [64], sorption [65] and swelling [66]. It is also noteworthy that 3D printers equipped with multiple printheads are capable of printing complex systems composed of various types of hydrogels which is a huge advantage compared to the traditional methods.

Karakurt et al. [35] successfully manufactured solid dosage hydrogel loaded with ascorbic acid with different geometries for personalised applications. The hydrogel was printed via SLA. A novel biocompatible photochemistry was used to obtain a custom tablet consisting of ascorbic acid (AA) encapsulated in a poly(ethylene glycol) dimethacrylate (PEGDMA)-based polymer network. Riboflavin was used as a safe alternative to toxic photoinitiators [67]. Different shapes were created, including coaxial annulus, and honeycomb patterns. The ability to manufacture various shapes demonstrates the precision of SLA printing and the potential of designing controlled-release formulations. The precise geometry and drug release profile tailored according to the individual needs meet the needs of personalised medicine for paediatric individuals, who tend to exhibit challenges with therapy adherence due to personal preferences and sensitivities. The obtained hydrogel tablets were checked in release studies for 6h. After one hour, the honeycomb and coaxial annulus-shaped hydrogels presented better release rates at approximately 80% of the initial amount of drug present (cumulative release) compared to the other shapes. It exhibits a chance for tailoring SLA gels for specific therapeutic needs, rapid or sustained release [35], which would be excellent in paediatric dosage forms.

**Figure 4:**
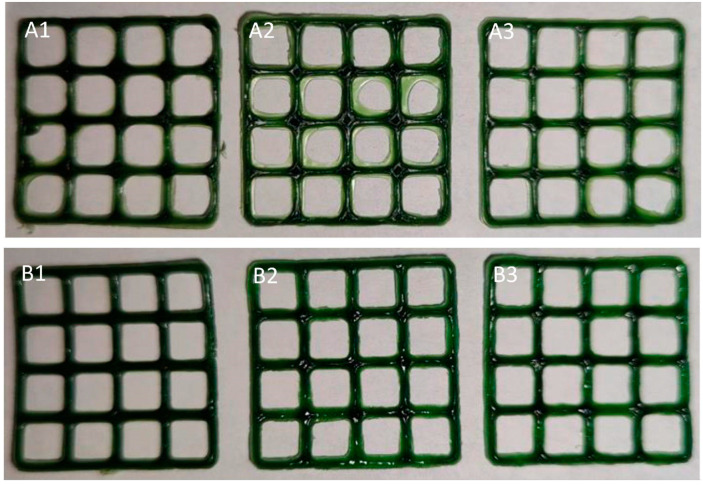
SSE 3DP lattices obtained from the aqueous PEO-EE gels: from gel 9 (**A1**–**A3**) containing 10 mg/mL of EE, 20% PEO and from gel 10 (**B1**–**B3**) containing 20 mg/mL of EE and 20% PEO (**B1**–**B3**) [68].

**Figure 5:**
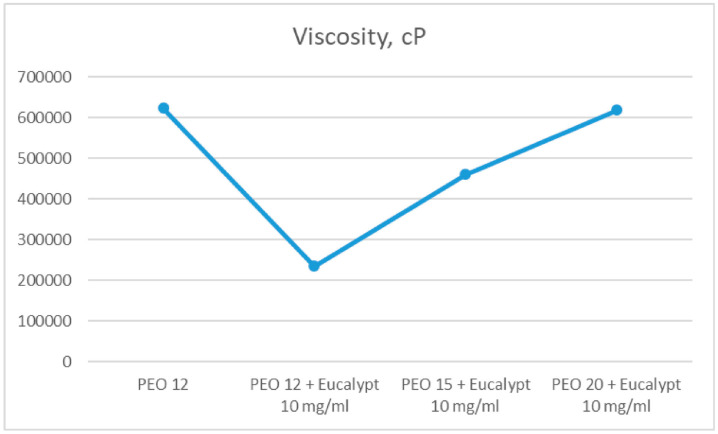
Changes of viscosity in the PEO gels (12%, 15% and 20%) loaded with the eucalypt extract in the dose of 10 mg/mL. [69].

In 2023, Koshovyi et al. obtained an SSE-printed eucalypt extract-loaded polyethylene oxide (PEO) gel for staphylococcal infections (Figure 4) [68]. It was manufactured as an alternative to the marketed anti-staphylococcal herbal liquid preparation, “Chlorophyllipt”, composed of an ethanolic eucalypt extract (EE), popular in Ukraine [69]. The aim was to deliver customisable DDSs with antimicrobial activity, suitable for the management of oral cavity infections and wound healing [68]. Chlorophyllipt is a 20% ethanol solution diluted with water at a 1:5 ratio, which is not a standardised procedure and has proven to be safe for children [69,70]. Ten formulations were prepared with different amounts of EE, eumulgin, ascorbic acid and PEO in gels. The composition of the investigated formulations displayed different viscosities (Figure 5). The final formulation with 20% PEO, 10 mg/mL EE, 30 mg/mL eumulgin, and 20 mg/mL ascorbic acid showed the best performance in SSE 3D printing. Thanks to higher PEO content, the viscosity, printability and structure were improved. Disintegration studies were carried out and it confirmed rapid dissolution around 10 min, which could result in classifying this gel as an immediate-release dosage form.

## 4. 3D-Printed Buccal & Mucoadhesive Films

Films are conventionally produced using hot melt extrusion (HME) and solvent-casting methods [71,72]. The latter method is simple and reproducible, but the most important drawback of this technique is the risk of air bubble entrapment and the possibility of changes in the film’s mechanical properties. On the other hand, hot melt extrusion is a continuous, solventless process. However, HME is unsuitable for thermosensitive drugs [45]. Solvent casting requires time since it usually consists of multiple steps, including the drying period, which can last up to 48 h [71,73,74,75]. On the other hand, films obtained with both of those methods need to be later manually cut to provide the desired dosage and size, whereas the 3DP method can use CAD to adjust the shape to the specific patients’ needs.

However, in novel soft dosage forms, films are predominantly obtained through SSE printing [47,68,76,77]. These films represent promising solutions for paediatric patients, characterized by their ease of swallowing or mucoadhesive and swelling properties. There are different types described in the scientific literature. In this review, first, we will focus on buccal films and mucoadhesive films and later on orodispersible films. Furthermore, dosage adjustment can be accomplished efficiently and swiftly due to the capacity to customize the film through cutting. It is essential that the film maintains homogeneity to ensure that the active pharmaceutical ingredient is uniformly distributed throughout its entirety.

### 4.1. Buccal Films

In 2024, Chachlioutaki et al. [76] worked on antifungal buccal films (BFs) to obtain an alternative to commercially used antifungal oral gel. BFs containing antifungal drug, miconazole, were printed with an SSE printer. The formulation contained a zein-polyvinylpyrrolidone (zein-PVP) polymer blend used as a drug carrier, and as a flavour enhancer, banana flavour drops were added to improve paediatric patient compliance. Different ratios of zein-PVP were examined, including 60/40, 50/50 and 40/60. For all films manufactured, disintegration time was determined to be less than 10 min, which is longer than most available oral gels. It was crucial to prolong contact with buccal mucosa, to increase the bioavailability and effectiveness of treatment of localised disorders [78]. BFs were investigated for mucosal adhesion and it turned out that the formulation containing zein and PVP at a 40:60 ratio had shown the strongest binding potential. Studies between BFs and commercially available oral gel demonstrated insignificant differences in cumulative and total drug penetration. Then, in the stability test, miconazole in the films remained stable after two months of storage. Additionally, changing the form of the drug from a gel to a 3D-printed film offers the advantage of a lower probability of side effects, due to the selective distribution of miconazole in studied films, as opposed to an oral gel [76]. These results show promising solutions for application in the paediatric population.

In 2024, Krishnan et al. [77] utilized the SSE printing technique to obtain buccal films with incorporated nanoparticles with the anti-HIV drug, dolutegravir. Polymer ink was composed of polyvinyl alcohol and sodium alginate. Antiretroviral therapy recommended for children involves the application of syrup or crushed tablets which is particularly challenging for developing countries, where fixed-dose combinations may not be available [79]. The films containing chitosan nanoparticles with dolutegravir prepared by spray drying had shown improved dissolving. The nanoparticulate form of the drug was attributed to the improvement of its bioavailability. Chitosan induced the film swelling in a simulated salivary fluid. In the dissolution test, 100% of the API was released during 4 h. The drug incorporated in the films showed high permeability through the buccal mucosa, which is a promising result in terms of employing this delivery route in paediatric antiretroviral therapy [77].

**Figure 6:**
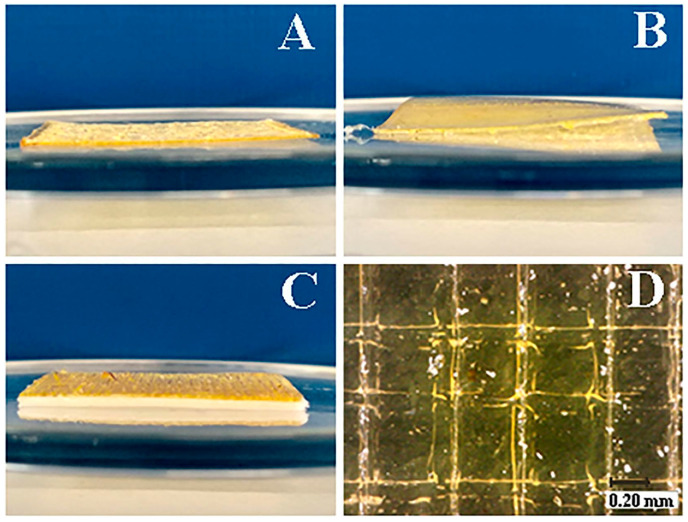
Representative photos of 3D printed films in the absence (**A**) and presence of ethyl cellulose (**B**) or wafer (**C**) backing layers. (**D**) Optical micrograph of 0C-X formulations (without chitosan, without backing layer). Reprinted with permission from [80].

Eleftheriadis et al. [80] worked on an alternative approach for the development of mucoadhesive buccal films (Figure 6), using the FDM printing technique as a timesaving and flexible method. The formulation consisted of polyvinyl alcohol (PVAl), which has been reported to present the desired properties for film forming and provide excellent printability, due to its thermoplastic features. Moreover, the composition of the buccal films included chitosan (CS) and a backing membrane composed of ethyl cellulose (EC) and triethyl citrate (TEC) applied as a plasticizer. The active layer of the film included diclofenac sodium (DNa) as the API and xylitol (Xyl) as a plasticiser for PVAl. Two drug-loaded filaments with differing compositions and an additional EC filament for the backing effect were produced using a single screw extruder. This resulted in the development of different formulations, as the backing layer could be present, absent or replaced with wafer paper edible sheets.

The fabricated products displayed satisfactory structural attributes and uniformity in dosage. Moreover, further characterisation indicated the amorphization of the drug, effective plasticization of the polymer and thorough blending of the components.

### 4.2. Orodispersible Film (ODF)

Lately, ODFs have been suggested as a DDS in the personalization of the therapy, particularly for children [81,82], as a result of the ability to combine the advantages of solid dosage forms and liquids. Musazzi et al. [83] developed ODF with maltodextrins with hot melt ram extrusion 3DP using a modified cartesian FDM 3D printer. Numerous different formulations were developed, consisting of maltodextrins (MDX) with a dextrose equivalent of 6 (MDX6) and 12 (MDX12), glycerine (GLN), glycine (GLY) and titanium dioxide (TiO_2_). Drug-loaded ODF included paracetamol, while the proposed method of preparation involved mixing the API with MDX and other excipients, the process of wetting the powders with glycerine and printing. Prepared ODF had a thickness between 150 and 250 μm, which was considered suitable for patients’ handling. Furthermore, the formulation dissolved within 3 min, complying with the European Pharmacopoeia (PhEur). Specifications and the uniformity of dosage units were also within the limits of the PhEur. The results of the dissolution testing confirmed the ability of maltodextrins to improve the dissolution rate. The authors considered this method of ODF development as prospective for further use.

**Figure 7:**
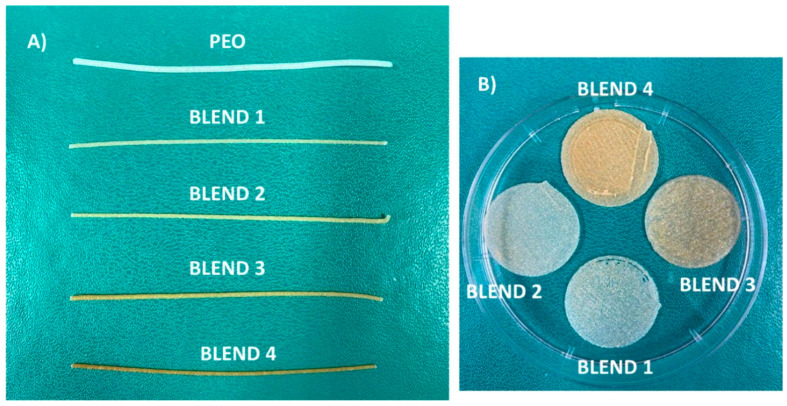
Filaments produced from the four different blends (**A**) and printed films (**B**). Reprinted from [84] under the terms of the Creative Commons CC-BY license.

Racaniello et al. obtained orodispersible mucoadhesive films (Figure 7) [84] to treat oral lichen planus (OLP), a chronic mucocutaneous disorder of the stratified squamous epithelium affecting the oral mucous membranes. Clobetasol propionate (CBS) is a corticosteroid commonly used as an API in the topical treatment of OLP [85]. Mucoadhesive tablets have been used recently to treat off-label OLP. This disease is rarely reported in the paediatric population [86]. However, the commonly accepted therapeutic approaches are not suitable for paediatric patients, and, as the authors indicated, the off-label topical therapies, as well as the available research, do not offer any good solutions for the paediatric population due to the increased risk of swallowing the applied formulation [87]. To prepare the personalised drugs, the mucoadhesive films were produced via a 3D-printing technique called direct powder extrusion. To obtain the formulation, HPMC, chitosan (CS), hydroxypropyl-β-cyclodextrin (HP-β-CD) and PEO were used. Additionally, to improve the aqueous solubility of CBS, HP-β-CD was added [88]. Four formulations were prepared.

Blend 3 presented the highest mucoadhesion, associated with the higher CS content. The solubility of CBS was increased because of the incorporation of HP-β-CD and partial amorphization occurring in the printing process.

**Figure 8:**
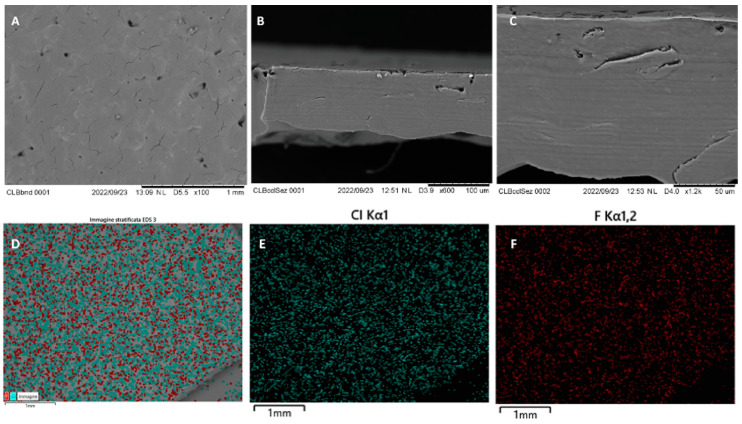
SEM images of the 3DP film surface (**A**) and cross-section (**B**,**C**). Surface chemical microanalysis of the printed mucoadhesive films (**D**) with images related to the presence of the elements Cl (**E**) and F (**F**). Reprinted from [84] under the terms of the Creative Commons CC-BY license.

Films obtained from blends 2 and 3 presented gradual disintegration, completed within 20–30 min. Stability studies were carried out and showed 3 months of stability under controlled storage. The films obtained by Racaniello et al. (Figure 8) are strong candidates for personalised dosage forms [84].

### 4.3. Oral Films and Fast Dissolving Oral Films (FDFs)

Roche et al. manufactured SSE caffeine (CAF) oral gel films with SSE for treating apnoea of prematurity based on stimulation of the respiratory centre in neonates [47]. Typically, caffeine citrate solutions are prepared and tailored to the paediatric patient based on body weight [89]. In this research for films, the extruding paste was prepared with hydroxypropyl methylcellulose (HPMC) and sodium alginate (SA) as a gelling agent Additionally, as a disintegrant, sodium croscarmellose (SC) was used to obtain a fast release. No research has found that these excipients are toxic to children. Different formulations were prepared, with low and high concentrations and at different ratios of gelling agents and disintegrants. Films were printed straight onto the Petri dish and were easy to collect. Only one formulation was too fragile to be handled due to complications during extrusion. Formulation 1C with 35% CAF, 52% SC, 8.2% SA and 4.8% HPMC had the best potential for commercial use due to the best printability, good swelling and no roughness detected. The oral films obtained in this study exceeded 2 mm in diameter, making them unsuitable for direct use in neonates. However, they could be administered after dispersion in a small amount of water or milk, commonly used as excipients in these departments. In further work, mucoadhesive properties should be investigated, and the disintegration time of the films should be improved. The investigated systems showed promising results for the formulation of caffeine-loaded gel films for infants.

Another study with paracetamol, which is the most widely used drug in the paediatric population [90], was conducted by Ehtezazi et al. [91], who produced FDF with taste-masking layers by FDM 3DP. The developed formulation included PVAl, polyethylene oxide (PEO) Mw 100,000 Da, PEO Mw 200,000 Da, poly (ethylene) glycol (PEG) Mw 4000 Da, and PEG Mw 30,000, starch, sodium starch glycolate, croscarmellose and sodium lauryl sulphate. The produced FDFs featured one of the following APIs, paracetamol or ibuprofen, and freeze-dried strawberry powder was used for taste-masking. Firstly, the excipients were ground together with the drug using a pestle and mortar to form a fine powder and then mixed for 15 min. Next, the entire mix was extruded by a single screw extruder to obtain straight filaments. Different FDFs were printed using the FDM printer. The printing took 2 min for single-layer plain film, whereas the printing time was 30 s for mesh film. The thickness variation across the films was below 4%, the uniformity of contents was within the limits required by Pharmacopeia and the weight uniformity exceeded 97%, except for the formulation consisting of a plain multilayered film. Furthermore, only mesh films presented a short disintegration time and in vitro dissolution tests showed that the drug release rate may be increased by manufacturing them as mesh. However, the 3DP FDF presented a longer disintegration time than commercially available FDF or films prepared using the solvent-casting method.

The paediatric films obtained with 3DP techniques are summarized in Table 2.

## 5. 3D-Printed Gummies & Chewable Tablets

### 5.1. Gummies

Gummies, also known as chewable gels, are novel formulations that are becoming increasingly popular among the paediatric population. They are intended for oral use and should be chewed before swallowing. From the physicochemical point of view, gummies can be considered elastic gels, as their base components usually comprise gelling agents and water [92]. As a thickening agent, gelatin is most frequently used, however, there is a growing need for vegan alternatives and gelatin is sometimes being replaced by pectin or carrageenans [93]. There are also ingredients whose function is to enhance the patient’s acceptance. Sweeteners, including sugar or artificial components, and flavouring agents can cover undesirable tastes and develop the attractiveness of the formulation. The incorporation of oils requires also the application of emulsifiers, while coconut oil and corn starch can also be added to prevent gummies from sticking to each other. Gummies are elastic and retain their shape in the mastication process.

Drug-loaded gummies, also called drugmies [94], resemble popular sweets, thus evoking positive associations among children. They attract them with their unique look and pleasant taste, which makes this formulation very attractive in the context of searching for novel drugs targeted at the paediatric population. It is important to notice that gummies are already widely applied and commonly accepted as dietary supplements with vitamins, minerals and omega-3 acids. Therefore, in the search for novel therapeutic approaches for children, particular attention should be paid to the factors directly affecting the attractiveness to children. Taking into consideration the need to fully customize the appearance, taste, texture and other sensory properties of the dosage form, 3DP seems to be an extremely attractive technology compared to a moulding technique applied in industrial manufacturing.

In recent years, studies have emerged on the topic of using 3DP to produce personalized drugs in the form of gummies. In 2019, Rycerz et al. [38] used the e-3DP method to produce chewable Lego blocks, comparing one- and multi-step embedded 3D printing. In this case, the embedded phase contained paracetamol and ibuprofen, and the embedding phase was a gelatine-based matrix. The multi-step version of the method appeared to provide better-quality printlets. During the chewing process, the gelatine matrix dissolves quickly, while the drug-loaded paste dissolves at a lower rate. In another two studies, a syringe-based extrusion 3D printer was used to produce gummies. Herrada-Manchón et al. [94] in 2020 manufactured ranitidine hydrochloride-based drugmies, investigating the effects of ranitidine and corn starch content on printability and formulation properties. Additionally, Rouaz-El Hajoui et. al. [95] compared 3D-printed hydrogels with dissolved omeprazole and omeprazole pellets. Here, the advantage of gels with pellets in the gastric resistance test was demonstrated. In the presented study, the need for novel formulation approaches for paediatric dosage forms with challenging drugs, including those susceptible to degradation in the acidic pH of gastric fluid, is highlighted.

**Figure 9:**
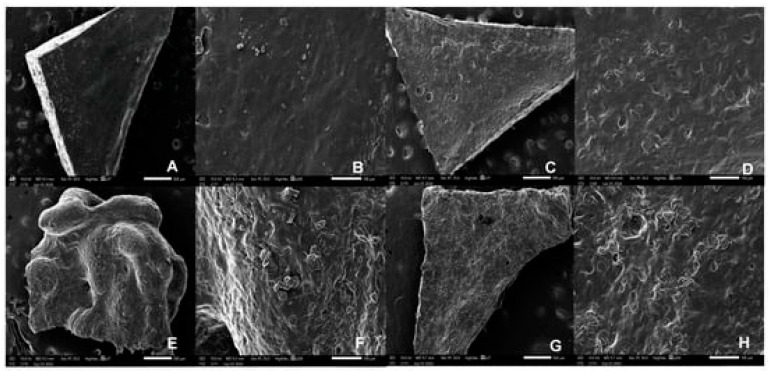
SEM micrographs of 3D printed gummies with and without MET at different magnifications. (**A**) 3D printed gummy surface with MET 37 × (500 µm); (**B**) 3D printed gummy surface with MET 200 × (100 µm); (**C**) 3D printed gummy middle layer with MET 37 × (500 µm); (**D**) 3D printed gummy middle layer with MET 200 × (100 µm); (**E**) 3D printed gummy surface without MET 37 × (500 µm); (**F**) 3D printed gummy surface without MET 200 × (100 µm); (**G**) 3D printed gummy middle layer without MET 37 × (500 µm); (**H**) 3D printed gummy middle layer without MET 200 × (100 µm) [98].

Looking forward, an exceptionally popular method of 3D printing of this pharmaceutical form is semi-solid extrusion. Tagami et al. [96] tested gummy dosage forms with lamotrigine, focusing on the effect of ink composition on the key characteristics of the finished material. The formulation’s base was composed of gelatine, HPMC, reduced syrup and water. The presence of HPMC provided good printability at room temperature, while gelatine content affected the strength of the obtained product. The drug release studies revealed that the investigated dosage form released 85% of the API within 15 min of the test. Zhu et al. [97] also pointed out the impact of taste-masking substances in propranolol hydrochloride-loaded gummies with gelatine and carrageenan. Moreover, Santamaría et al. [98] analysed the difference between the formulation with SSE (Figure 9) and the convective casting methods. This study also demonstrated the possibility of using 3DP to produce novel dosages containing metformin (MET). As the previous papers noted, gummies are produced using gelatine, which is a zoonotic product.

Ganatra et al. [93] obtained a vegan-friendly product, with pectin as a gelling agent. Furthermore, this ingredient allows us to obtain low-calorie, personalized gummies containing simethicone.

**Figure 10:**
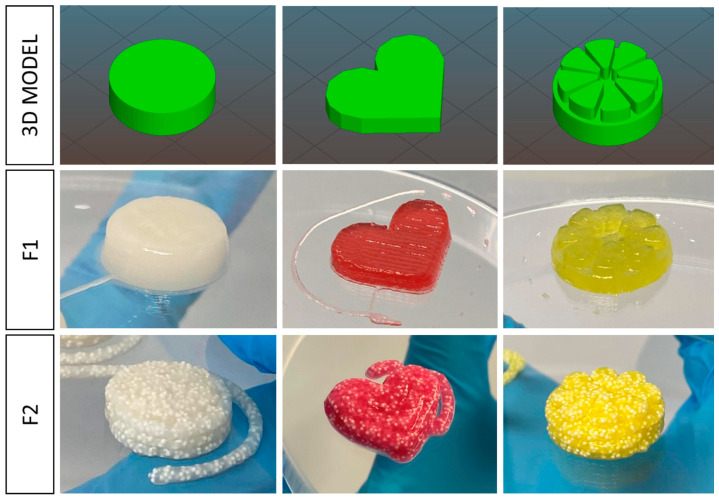
**3D models** and drugmies printed with formulations containing omeprazole powder (**F1**) and omeprazole pellets (**F2**) [95].

As highlighted above, the attractiveness of this novel formulation is highly significant. It can be achieved through an aesthetically appealing design, intriguing appearance, and pleasant taste qualities. In some papers, researchers presented shapes adapted to the paediatric population and examined the printing possibility and quality of the prints. Other considered aspects were the addition of food colouring and flavour enhancers. There has been a strong focus on organoleptic properties in studies of propranolol gels [97]. Zhu et al. designed shapes of capsules, diamonds, flowers and bears using different compositions to decide which one would work the best. The authors also checked the impact of sucralose and bitterness inhibitors: γ-aminobutyric acid and ferulic acid. Ten healthy volunteers evaluated the taste of preparations, after chewing without swallowing. The study revealed that increasing only the concentration of sucralose is not enough for a satisfactory result. The best medicine taste-masking effect was obtained by combining the drug with γ-aminobutyric acid. In another paper [94], researchers presented disc-, heart- or bear-shaped gummies. The attractive appearance was obtained with food colouring responsible for reddish tones, and corn starch, which caused a whitish-pastel shade. Flavour improvement was assured by adding strawberry essence and liquid sweetener. All printed gummies were defined as visually identical and with high fidelity to the project. No undesirable spots or particles were seen. Gummies filled with pellets have appeared in the shapes of disks, hearts and lemon slices (Figure 10) [95].

**Figure 11:**
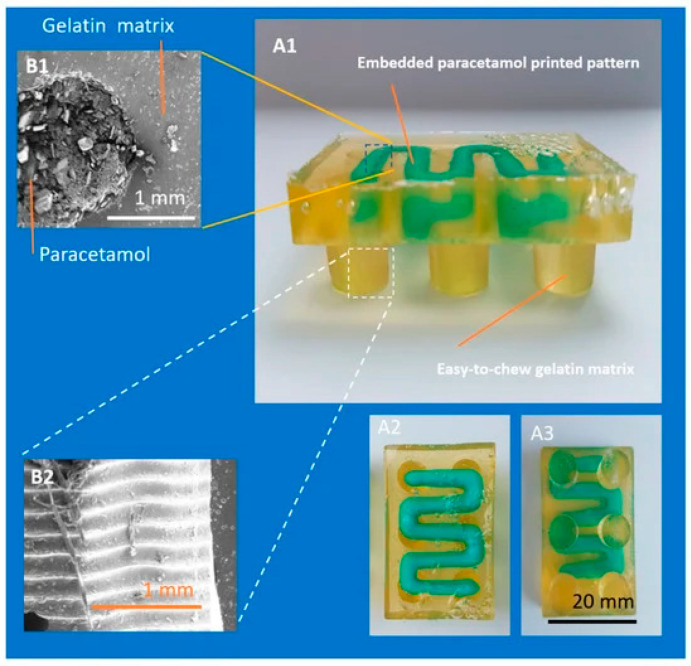
Photographs of (**A1**) side, (**A2**) front, and (**A3**) back view of Lego™-like soft gelatine with embedded paracetamol dose. SEM images of (**B1**) cross-section embedding and embedded matrix and (**B2**) surface of gelatine-based matrix [38].

They were described as shiny and colourful, with good appearance and smell; however, pellets were clearly visible. Interesting shapes that could also be found in studies are square, star, diamond, pentagon, heart, cylinder, triangular, hemisphere, doughnut [96] and Lego blocks (Figure 11) [38].

**Figure 12:**
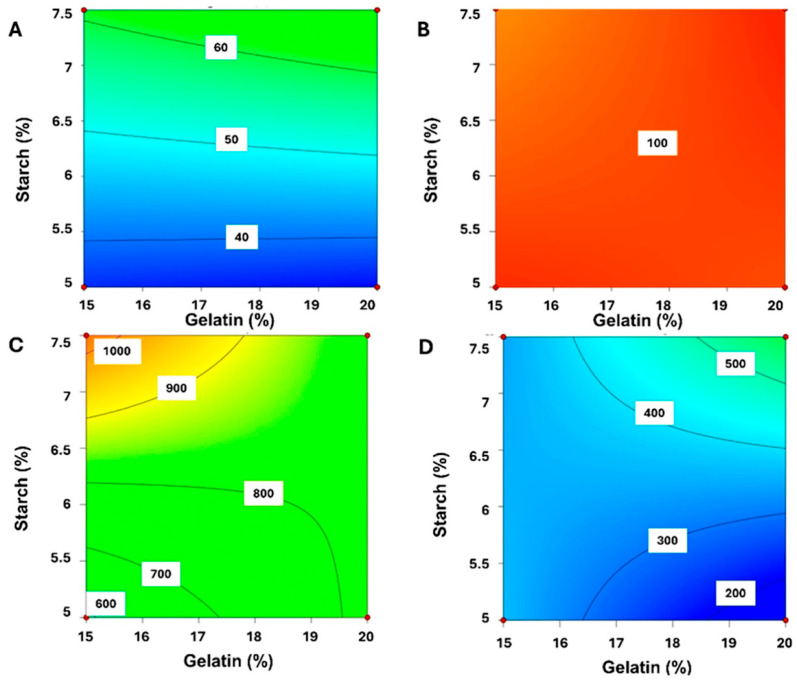
2D Contour plots for optimization of gummies with metformin. Drug Release (**A**,**B**); Firmness (**C**,**D**). The effect of 3D printing on firmness and drug release is represented in (**A**,**C**) while the effect of casting is illustrated in (**B**,**D**) [98]. The colours correspond to the values represented by the lines (blue: the lowest values, red: the highest values).

Among the data presented in studies, one can see the influence of glucose and other components on the properties of the formulation. Viscosity is a key parameter in deciding about printability. If it is very low, ink leaks from the nozzle too easily, but if it is very high, much pressure would be needed for extrusion. According to the available literature, rheological studies can provide valuable information on the properties of the semi-solid mass prepared for gummy extrusion. Tagami et al. prepared and examined formulations with differing compositions using a cone-plate rotational geometry viscometer. The aim of the performed tests was to find an optimal amount of the ingredients that should be used for the best results. Increasing both gelatine and HPMC contents increased the viscosity of the ink. However, HPMC content did not affect hardness. The properties that also depended on gelatine concentration included strength [96], toughness, springiness and chewiness. Moreover, gelatine content decreased adhesiveness and at the same time increased the cohesiveness of the gummy. Carrageenan reduced printability and elasticity. Nevertheless, together with gelatine, it had a positive influence on complex modulus (G*), which is a measure of the resistance to deformation of the formula [97]. Starch content relative to gelatine raised the firmness in 3D-printed forms, while for casted gummies, the higher the gelatine content, the greater the firmness (Figure 12) [98].

Also, using corn starch had a good impact on storage modulus values, resulting in better self-supporting ability and higher mechanical strength of the ink [94].

### 5.2. Chewable Tablets

Chewable tablets belong to oral dosage forms, whose function is to produce a palatable residue in the oral cavity and facilitate swallowing. United States Pharmacopeia (USP) distinguishes two types of chewable forms. Some can be chewed for ease of administration, but it is not required. However if the word “chewable” is included in the formulation’s name, it must be chewed or crushed before swallowing, to avoid choking and enable proper release of the API [99,100,101,102].

Traditionally soft chewable tablets are prepared by moulding or extrusion process, but as it is a preferable form for paediatric use, it is essential to enable individualization of drugs administered in this way. 3DP is a method eagerly studied in this area [99].

**Figure 13:**
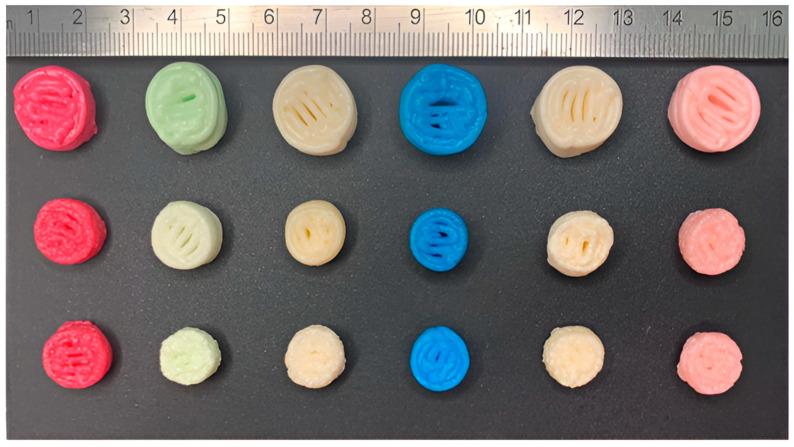
3DP chewable tablets printed in different colours and flavours. Reprinted from [104] under the terms of the Creative Commons CC-BY license.

**Figure 14:**
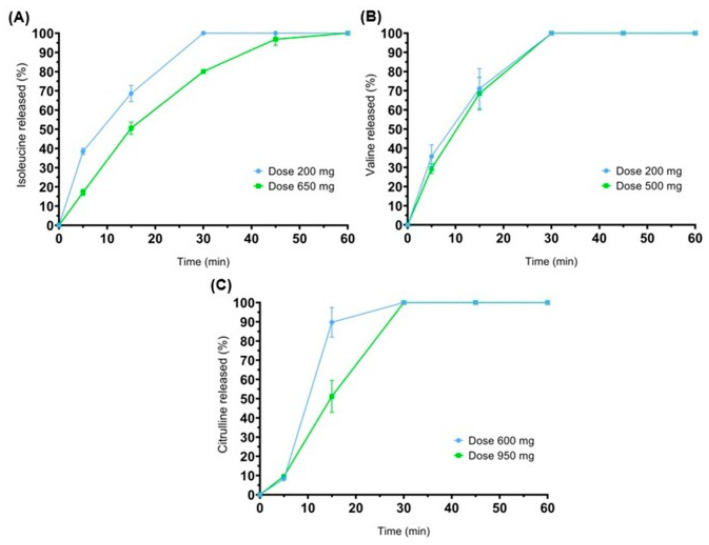
Release profile from: (**A**) Isoleucine; (**B**) Valine; and (**C**) Citrulline chewable printlets. The blue line represents the lowest dose, and the green line the highest dose (n = 3). Reprinted from [104] under the terms of the Creative Commons CC-BY license.

One of the examples describes chewable tablets for potential use in a rare genetic disease. Maple syrup urine disease (MSUD) is a metabolic disorder, affecting 1 in 185,000 newborns. Patients have to supplement isoleucine and valine, and the dosage should be adjusted for age, weight and lab results. Two similar studies about using 3D-printed chewable tablets to treat children with MSUD, both being prospective, single-centre and crossover, are available in the literature. Firstly, Goyanes et al. [103] made chewable tablets containing isoleucine by SSE printing. Gel-based ink included pectin, sucrose and maltodextrin. Six types of different flavours and colours were designed to see children’s preferences. Four patients aged 3 to 16 took part in the research. Later, Rodríguez-Pombo et al. [104] published another paper about SSE-printed MSUD chewable tablets, consisting of pectin, sucrose, maltodextrin, water and maltitol (Figure 13). This time, more amino acids were tested (citrulline, isoleucine, valine) and they made isoleucine and valine combinations in one tablet. The release profiles of the applied actives are depicted in Figure 14.

Also, new flavours were evaluated. Six patients were subjected to the experiments. Both studies focused on comparing the efficacy and acceptability of 3D-printed chewable tablets to conventional medication. All the patients maintained controlled optimum amino acid levels. Patients or their parents have evaluated the formula and flavours. The results showed a high level of acceptance, especially about orange, vanilla and lemon flavours.

Another study has been performed to develop a treatment for kids with adrenal insufficiency, characterized by low cortisol levels [105]. The therapy is based on cortisol substitution. Because of the lack of dosage forms compatible with paediatric patients, attention has been drawn to the need to develop children-friendly, easy-to-individualize formulations. The aim of the project was to optimize the SSE-printing process of chewable hydrocortisone tablets that would be later used to conduct a clinical study. The ink was based on sucrose, pectin, maltodextrin, water and maltitol. To meet the requirements of children, flavouring and colouring agents have been included among the ingredients. The results show immediate hydrocortisone release and good stability for 1 month of storage.

SSE has been also used to produce chewable amlodipine tablets, to help with paediatric hypertension problems [106]. To validate the final composition, formulations with different concentrations of carboxymethyl cellulose sodium (CMC-Na), sodium starch glycolate (SSG) and glycerine, have been prepared. In the results, its influence on the tablets’ properties can be seen. High hardness is not a desirable feature as it can decrease palatability. CMC-Na content is positively correlated with hardness, which is opposite to SSG and glycerine contents. These factors also affect friability, which can be reduced by the increasing CMC-Na and SSG contents. It can also be reduced by glycerine, thanks to its wetting features. Importantly, CMC-Na turned out to be the key factor that determined 15 min dissolution. As the formula is dedicated to kids, bitterness inhibition was examined, leading to the selection of sucralose and lemon essence as masking agents.

The most important details regarding the studies on gel-based 3D-printed paediatric dosage forms are summarized in Table 3.

## 6. 3D-Printed Chocolates & Novel Delivery Forms

### 6.1. Chewing Gums

Synthetic chewing gums were first developed after World War II, however, prior to its development people chewed natural, plant-based gums [107]. Although well-known for its confectionery role, chewing gum plays a vital role as a drug delivery system [108]. European Pharmacopoeia (Ph. Eur.) defines medicated chewing gum (MCG) as “solid single dose preparations with a base consisting mainly of gum that are intended to be chewed but not swallowed, providing a slow steady release of the medicine contained” [109]. The Japanese Pharmacopeia [110], USP [111] and Ph. Eur. mention the application of MCG as an important drug delivery system. They are regarded as a relatively recent drug formulation for both transmucosal and oral drug delivery [112]. From MCG, the drug is gradually released during chewing and can be swallowed with the saliva causing systemic effect or can be absorbed via the buccal mucosa acting locally [113]. This DDS is designed to be chewed between 10 and 30 min, allowing the API to dissolve in the saliva [114]. Nevertheless, the rate and pattern of chewing are also vital factors affecting the release of API from the formulation [113].

Currently, MCGs are the first choice of patients for nicotine replacement therapy [115] and there is a substantial amount of new research on the application of chewing gum in healthcare. The literature reports on chewing gum as a DDS for numerous substances, such as metformin, clotrimazole, miconazole, cetirizine, domperidone maleate, levocetirizine and caffeine [114,116,117,118,119,120,121], also including plant-derived substances like curcumin or essential oils [122,123,124].

Elastomers are the key ingredient in the base of the gum; they determine cohesiveness and rubbery texture. High concentrations provide toughness, whereas gums containing less elastomers are also less elastic [125]. Common elastomers include styrene–butadiene (SBR) and polyisobutylene (PIB) rubbers; also, polyvinyl acetate (PVAc) can be used to balance the plasticity and elasticity.

Chewing gums offer numerous advantages for paediatric drug delivery, starting with the ability to administer the drug anywhere and anytime, without the intake of water. Additionally, MCG provides high patient compliance, since it is easy to mask bad or bitter tastes and there is no need to swallow; this trait is especially important for patients with dysphagia or children. Furthermore, this formulation allows easy self-medication, without drawing any attention to the treatment, providing stigma-free curation [113]. Furthermore, additional positive aspects of chewing gum are mentioned, such as stress relief [126,127], improved weight management [128] and oral care [129].

While there is a substantial amount of research regarding MCG, there is limited insight into the topic of 3D printing of chewing gums. Literature review articles [130,131,132,133] mention Alec [134] or Krassenstein [135], describing the research of two students from London, who developed the prototype GumJet 3D printer. This innovation features a cartesian-style 3D printer with *x,y* and *z*-axes and a platform which extrudes the gum filament out of a thin metal extruder, resembling the commonly used FDM printers. The invention allows the production of personalised chewing gum consisting of gum resin and flavouring with interesting texture, due to its layer-by-layer production process. Unfortunately, there is no further information on the project.

Another approach to developing chewing gum using the 3DP technology was presented by Wacker Chemie AG and their CAPIVA^®^ 3D technology. This product was tailored specifically for printable gum, to enable the development of chewing gums in various shapes according to the consumer needs. Wacker also issued a patent application on the chewing gum composition and method of shaping chewing gum in a 3D printer. The invention features a composition of maltitol, mannitol, PVAc, vinyl acetate–vinyl laurate copolymer, gelling agent, emulsifier and water. Moreover, the method of printing by heated cartridge in a heated print head is also a part of the patent application. However, the current status of the patent is abandoned [136].

The literature search highlights a niche worth exploring by researchers. It appears that merging the 3DP technique and medicated chewing gum formulation can be beneficial in terms of therapeutic efficacy and addressing the needs of special populations, including paediatric patients.

### 6.2. Chocolate

Chocolate is one of children’s favourite sweets. This is because of its pleasant taste but also fusible consistency. Thus, using chocolate as a drug vehicle can not only improve adherence but also influence children’s emotional approach to medical treatment.

The study of Karavasili et al. [137] presents a novel formula of chocolate and corn syrup-based, soft solid drug, produced with extrusion-based 3D printing. Paracetamol and ibuprofen were used as model drugs to test the ability to use this method for hydrophilic and lipophilic drugs. Chocolate is a dispersion of cocoa and sugar particles suspended in cocoa butter. Particle size distribution affected the sensory properties of the product. Large particles (above 30 μm) would be responsible for bad mouthfeel and grittiness. Mean particle diameters were 14.2 μm for ibuprofen ink and 28.1 μm for paracetamol ink, with some percentage of particles above 30 μm. Also, adding corn syrup contributed to a slight increase in the mean size of the particles. However, the parameter stayed below 30 μm, which indicates an acceptable quality. The peak melting temperatures for chocolate formulations with drugs were 32.4 °C and 29.1 °C, which makes them easily melt at the human body temperature. The beginning of the melting process was marked as 28.6 °C and 24.5 °C; thus, the final product should stay solid at room temperature. To match the product to paediatric patients, the 3D designs appeared as nice simple shapes and cartoon characters. Chewiness is another important factor determining the acceptance. Adding syrup reduced the hardness of the formula and increased viscosity and adhesive properties. The dissolution tests performed in simulated salivary fluid for the chocolate-based sausage forms resulted in fast and high-level dissolution in the oral cavity environment.

Later, Lesutan et al. [138] examined the impact of using soy lecithin as an emulsifier. The effect of lecithin on chocolate viscosity was compared to the study investigating the effects of corn syrup by Karavasili et al. [137]. It was found that the chocolate-based dosage forms with lecithin were characterized by lower viscosity. Moreover, soy lecithin increased the firmness of the chocolate product.

Chachlioutaki et al. [139] compared chocolate-based dosage forms obtained with 3DP and conventional, mould-casting method. Paracetamol was used as a model drug in this study. 3D-printed chocolates were designed as cuboids in different sizes and produced with SSE technology. 3D-printed forms were shaped and sized according to the design and were free of structural irregularities observed in the moulded dosage forms. The melting maximum point of the 3D-printed products was determined to be 32.5 °C. Mass uniformity was verified according to the Ph. Eur. 3D-printed forms have met the requirements, as percentage variations did not exceed 5%. The moulded dosage forms showed greater fluctuations, failing the mass uniformity test. Paracetamol content uniformity was also examined, with the result of 3D-printed chocolates within acceptance value and moulded chocolate failing to pass the acceptability criteria. Moreover, 3D-printed dosage forms were found to be softer.

Milliken et al. [140] investigated honey- and cocoa-based formulations as carriers for vitamin D_3_ (VitD3). The study aimed to assess the potential of three types of raw Greek honey in enhancing the palatability and functionality of printlets. To achieve this, different ink formulations were prepared, each containing one of the three honey varieties—Vital honey (VH), Lavandula angustifolia honey (LH), or Jerusalem sage honey (JH)—at concentrations of 5% or 10%, with or without added VitD3. Thermal analysis confirmed the successful dissolution of honey and VitD3 into the cocoa vehicle, enabling further evaluation of their impact on formulation properties. In texture analysis, the deformation of the printlets was measured in terms of force (N) and distance (mm). Considerable variations were observed, with force values ranging from 0.13 ± 0.05 N to 11.86 ± 0.74 N and deformation distances from 0.22 ± 0.17 mm to 2.31 ± 2.46 mm. While statistical analysis indicated no significant differences, suggesting that the ingredients did not strongly influence force and distance measurements, a general trend was noted: JH and LH tended to increase firmness. Conversely, the addition of VitD3 to JH formulations led to reduced firmness and increased deformation distance, highlighting the need for further investigation. Nonetheless, the authors suggest that honey and VitD3 could play a crucial role in determining the final firmness and elasticity of the printlets. The results of rheological analysis were inconclusive, though most printlets exhibited decreasing viscosity with increasing shear rate. In disintegration tests, all printlets successfully disintegrated within 30 min, with formulations containing higher honey concentrations taking slightly longer to break down than those with 5% honey content. Additionally, VitD3 further extended the disintegration time. Since honey improves taste while also delaying disintegration, printlets with honey-enriched formulations are expected to be more palatable and better accepted by children.

The essential details regarding the literature examples of 3D-printed non-gel paediatric formulations are summarized in Table 4.

**Figure 15:**
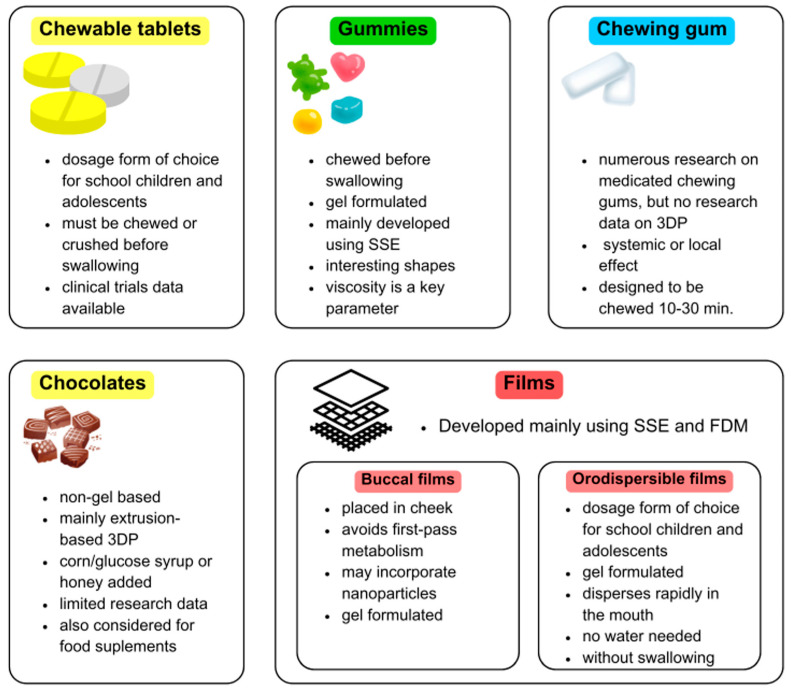
Summary of 3DP soft dosage forms for paediatrics.

The most important details regarding paediatric formulations obtained with 3DP techniques are summarized in Figure 15.

## 7. Challenges and Benefits of 3DP in Paediatric Drug Development

### 7.1. The Role of 3D Printing in Addressing Challenges of Paediatric Drug Formulations

The development of modern technologies and solutions using 3D printing methods is progressing rapidly, making it necessary to look at the challenges of various formulations and drug forms. Particular requirements are made, especially for paediatric drugs, taking into account that the paediatric group is not representative, but divided into smaller subgroups, including neonates, preterm newborns, newborns (0–28 days), infants (over 28 days–12 months), toddlers (over 12 months–23 months), preschool children (over 2–5 years), school-age children (6–11 years) and adolescents (12–18 years) [141]. Paediatric formulations must be tailored to each age group because of the different biochemical activity of enzymes compared to adults, as well as the alteration of intestinal transit or higher pH of gastric fluids [142]. Considering that children’s organs and physiological systems are still maturing, the absorption and distribution of API may differ significantly from those in adults [143]. In addition to the mentioned aspects, the precise dosing is an important issue. Taking into consideration factors such as the child’s weight, age, volume of the measured dosage form, ease of administration and acceptability of taste, it is possible to meet the individual needs of such a patient [144]. Aligning with these specific norms increases the chances of effective therapy and reduces the risk of side effects due to possible overdose. The immediate need for adjusted formulations in the treatment designed for paediatric patients is a pressing concern. It is important to note that 3DP also allows for quick adjustment of the therapy in terms of dose or drug release profile which may be beneficial for therapeutic efficacy whenever a quick intervention is required. It is of particular importance in paediatric patients. Furthermore, additive manufacturing compared to finished medicine production may become handy for prototyping dosage forms even though the end product may be manufactured with a different method [145]. Dose individualization is one of the greatest challenges of contemporary pharmaceutical technology. The commercially available pharmaceutical products are obtained in mass manufacturing processes, according to the frequently mentioned in the scientific literature paradigm “one size fits all” [146]. It is obvious that industrial production is fast and cost-efficient, allowing for obtaining numerous single drug doses in a short time with good manufacturing process repeatability. However, this universal approach is not always the best one considering the therapeutic effect, especially in a paediatric population characterized by high body mass and metabolic enzyme activity variability. Although the process of dose individualization is challenging, it is an inherent advantage of 3D printing drug manufacturing and is its differentiation from traditional technologies. This is especially true for the production of drugs with narrow therapeutic indices. This innovative approach opens up many new opportunities in pharmacotherapy, especially in areas that require precision and flexibility, and allows for the implementation of customized treatment plans [147].

### 7.2. Cost Effectiveness and Scalability

Commercialization of 3DP causes high costs of developing new formulas or re-developing formulations suitable for the printers, selecting and buying new excipients and maintaining the pharmaco-technical properties of the final product [148]. The 3D printing technique is focused on obtaining small batches rather than big-scale production. Taking into consideration the flexibility of 3DP in terms of the possible drug dose and release profile adjustments, it seems to be an ideal tool for therapy individualization. However, due to the character of this technique, it can be primarily used in small-scale production. Time constraints make other, traditional drug manufacturing technologies much better suited for large-scale production due to their more extensive infrastructure. Employing 3DP in industrial manufacturing would require expanding the facilities of the 3D printing area with more efficient solutions and that would involve an even greater increase in manufacturing costs and additional logistical problems [72]. Therefore, taking into consideration the potential economic aspects of 3DP-based products, it seems that they will be more expensive than their mass-produced alternatives obtained with traditional methods. An increase in expenditures is also associated with the need to use strictly defined excipients, which are selected on the basis of either inactive ingredient database (IID) or generally recognized as safe (GRAS) category. On the other hand, the same excipients must comply with the requirements of the applied 3DP technique, i.e., display specific thermoplastic properties or the ability to crosslink upon contact with UV light. This makes these methods non-universal and different in terms of financial outlay.

### 7.3. Spatial Arrangement

Regarding the production of small batches of material, 3DP provides a convenient adaptation to small production spaces, which is a favourable solution for manufacturing small batches of product. 3DP of medicines at the hospital effectively addresses the problem of drug supply discontinuity and shortages of medicines in hospital pharmacy stock. With the potential to produce personalised medicines in-house, patient needs can be met regularly, eliminating the dependence on drug delivery on external suppliers and potential delays in the supply chain [149].

### 7.4. Toxicity Considerations of Excipients—Processing Issue

Consistently, the toxicity of material used for printables needs to be taken into account. There are many different literature positions on using such excipients in oral dose treatment. For example, there are multiple statements about polyvinylpyrrolidone (PVP), which is used in printing and in traditional manufacturing methods as a binder for oral dosage forms. It is also applied as a food additive. The U.S. Food and Drug Administration (FDA) states that maximum daily exposure is between 15 and 60 mg. There are no studies on paediatric patients with PVP as an ingredient; therefore, the safety situation in the paediatric population remains unclear [144]. The most popular excipient for FDM is polyvinyl alcohol (PVAl) which requires relatively high temperatures to be extruded in the 3DP process. However, the problem might appear if the drug subjected to extrusion with the polymer is thermolabile, like 4-ASA and levetiracetam [150]. The techniques involving photosensitive polymers, like stereolithography (SLA) and laser sintering are rarely used in pharmaceutical applications and the materials suitable for these procedures are not included in the FDA’s Generally Recognized as Safe (GRAS) list which is a serious limitation in terms of the development of a pharmaceutical product. Additionally, some materials are attributed to severe drawbacks, including high costs, toxicity, unpleasant odours and the need for maintenance to prevent polymerisation [151].

### 7.5. Waste Management

Many common methods of producing pharmaceuticals are associated with the difficulty of processing manufacturing waste, which is an environmental problem. 3D-printing technology may be a favourable alternative due to its ability to produce small batches of potential drug dosages, associated with reduced post-processing residues. On the other hand, if systemic errors occur during printing and a batch of products turns out to be defective or unusable, there is a problem in terms of recycling. The potential use of 3D printing in the pharmaceutical industry does not allow for the reprocessing of used materials due to the possibility of losing certain properties, or even because of harmfulness. For these reasons, finding suitable solutions to the disposal problem is necessary [152]. Another important issue is energy consumption and carbon dioxide emissions. It has been shown that lowering the processing temperature of materials in 3DP can significantly reduce energy use and thus also reduce the generation of large amounts of CO_2_, which is a greenhouse gas notable for global warming. Unfortunately, heating and melting plastics used as starting materials in 3DP can also lead to the release of many volatile chemicals that are toxic to health. Getting them into the air poses a threat to the environment and humans, so it is essential to develop operational standards and compile new formulations that would not require high printing temperatures, as well as the use of an appropriate ventilation system containing standardized filters [149,153].

### 7.6. Standardization

Transformative applications have emerged in the medical industry, encompassing the production of medicines and personalised medical devices, highlighting the importance of emphasising concern for the repeatability of the 3D printing process [147]. To ensure the exceptional quality of each printlet and facilitate tracking software logs, digital fingerprints should be incorporated for each batch. The mentioned solution could align with regulatory expectations for traceability. Extending software used in 3D printing for editing projects should use previous errors from previous prints to learn via machine learning to help eliminate the defects and sustain the standard.

Continuous advances in 3DP technology mean that its techniques continue to improve. Products produced by these methods still show some defects in terms of quality. A common phenomenon is a recess at the base of the product, which is in the process of solidifying, and is caused by the weight of the rest of the model, known as an elephant’s foot. In turn, the characteristic formation of spherical structures during solidification is balling and occurs because of an insufficient surface tension of the material. The result of this appearance is that adjacent layers do not adhere to each other. Several methods also experience shrinkage during the final heating process, which manifests itself in a uniform or uneven form. However, some defects can be eliminated or prevented [60,153]. Taking into consideration the pharmaceutical aspects of 3D-printed dosage forms, it must be kept in mind that dose and drug release profile uniformity, as well as product stability, are the most important issues.

### 7.7. Ensuring Safety and Quality

The new and progressive solutions offered by 3D printing make certain demands on the technology, especially in matters of safety. For the 3D printing of drugs to be a future proposal for the production of marketed formulations, the excipients used for this purpose must be safe for patients. The photo-curable resins used in stereolithography, for example, are largely compounds containing toxic substances, and while they are well suited for structuring drug forms by 3D printing, they can be hazardous to health, ruling out the use of this technology for this purpose at present [35,144].

In addition, the 3D printing manufacturing process itself should be regulated in such a way as to maintain product reproducibility in terms of both the quality and properties of the drug form produced. This, in turn, poses the need to individualize the guidelines for each 3D printing method, since the same products manufactured using different technologies may end up having different properties, especially concerning mechanical strength and chemical durability [144].

Saydam and Takka [154], in their study on improving the solubility of rufinamide, a paediatric anticonvulsant drug, demonstrated that the dosage form obtained using 3D printing technology showed better solubility than the standard registered form. These results indicate that the 3D-printing manufacturing process itself and its steps affect the physicochemical properties of the final product. In order to ensure and sustain the reproducibility of these processes, it is necessary to look at these factors and systematize them accordingly [144].

Regrettably, the issue of regulating the manufacturing processes, the safety of the materials used and quality control within 3D drug printing is complicated and requires the development and implementation of appropriate solutions almost from scratch. Common quality control methods, which are suggested by the Food and Drug Administration and used in traditional drug manufacturing, are not always suitable for measuring the parameters of products manufactured with 3D printing technology. There is no universal procedure for ensuring and controlling the quality of pharmaceutical products produced by 3D printing. This is due to the fact that these quality controls and tests are mainly dependent on several factors, such as the shape and type of the product, the properties of the formulation components, and the printing parameters.

To this end, manufacturers should consider implementing a plan for dedicated quality control methods for this technology that takes into account their individual needs [144,155]. The laws and regulations for these innovative solutions need to be revisited and modified, taking into consideration the performance of modern 3D printers and software. Technological development and systemization of new processes to ensure the safety of 3D printed drugs, while challenging, are necessary for them to be approved by regulatory authorities and available to patients, especially paediatric patients. Low-quality products can be obtained fast with ease. Moreover, the lack of safety measurements gives a pass to possible counterfeits, to the detriment of patients [148].

**Figure 16:**
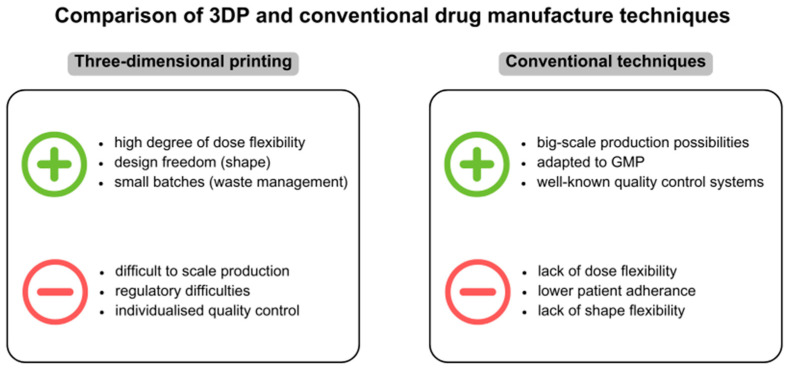
Comparison of 3DP and conventional techniques.

The comparison of the most important features of 3DP and conventional drug manufacturing techniques are presented in Figure 16.

## 8. Future Perspectives and Conclusions

There is a significant need to continue the development of medicines tailored to paediatric patients’ needs. The majority of drugs available on the market are not child-friendly; therefore, there is a need for pharmaceutical compounding or pharmaceutical manipulation in clinical practice. However, due to the rapid development in the 3DP technology, there are perspectives for significant change in the near future.

### 8.1. Research Trends

In recent years, interest in the 3DP technology increased, making it more cost-effective, widely available and affordable [156]. Moreover, scientific and technological progress drives economic growth, prompting all countries to invest more in research and development (R&D) [157]. According to the 2017 Forbes magazine [158], there is a noticeable increase in 3D printing spending, with 57% of manufacturing companies worldwide increasing their research and development investment in 3D printing. Furthermore, the 3DP technology is perceived positively since 95% of manufacturing companies regard it as a vital strength for enterprises in the market competition. The 3DP is also profitable since 47% of 3D printing companies have a higher return on investment than they did in previous years [159].

**Figure 17:**
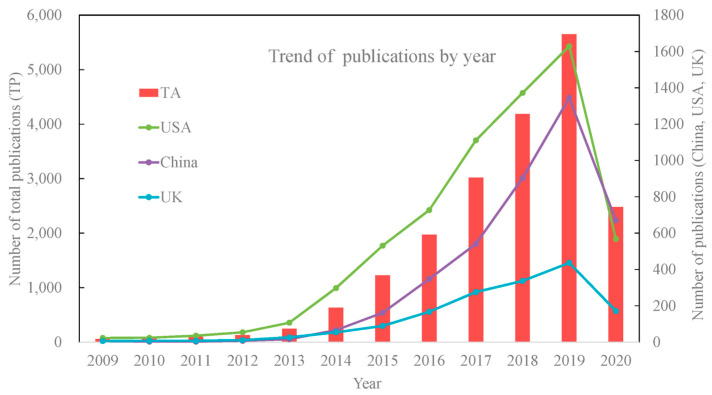
Trends in the number of publications related to 3D printing by year [161]. TA—the total amount of publications (left vertical axis). The vertical axis on the right corresponds to the number of papers published in China, USA and the UK.

Consequently, this growth in the R&D expenses on 3DP is followed by the visible trends in the number of publications on the topic (Figure 17) [160]. The drop in the number of papers published in 2020 is related to the methodology applied by the authors analysing the trends, as the data were retrieved in June 2020 and the last bar in the plot obviously does not provide the full number of papers on 3DP in this year.

**Figure 18:**
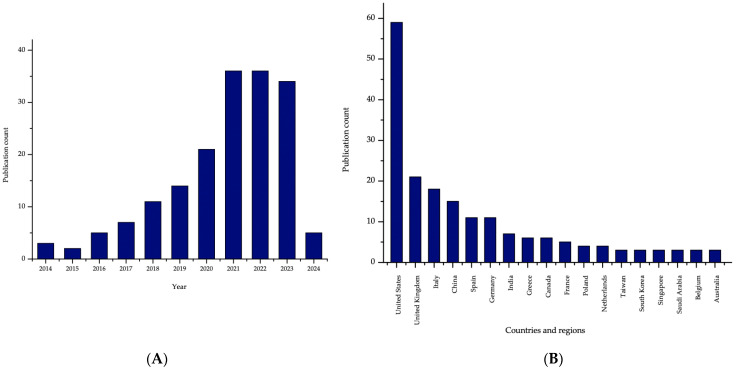
The number of journal publications relating to 3DP in the paediatric population from 2014–2024 (**A**). The number of journal publications relating to 3DP in the paediatric population in the top 18 countries (**B**) [48].

Additionally, the increase is also observed in the paediatric research on the topic of 3DP drug development (Figure 18). Again, the drop in the number of papers in 2024 is related to the methodology of the study.

### 8.2. 3DP & Patents

As 3DP becomes more widespread, the patent landscape also grows. Early patent applications connected to 3DP in the 1990s were related mainly to the rapid manufacture of prototypes and components [161], whereas more recent patents also focus on the medical field [162,163]. For instance, Orthopaedic Innovation Center, Inc. (Winnipeg, MB, Canada) focuses on the development of antimicrobial implants, fabricated using 3D-printing methods with WO 2014/075185 and WO 2015/143553 patent applications.

Furthermore, there is a limited number of published patent applications relating to the 3DP methods for direct production of pharmaceutical dosage forms, such as 3DP dosage form comprising a high dose of levetiracetam [164] or the broader range application by the University of Central Lancashire for the invention of fused filament fabrication 3DP for producing solid dosage forms, for example, tablets, the printing apparatus and more [165]. Further examples include the multiple layered oral dosage forms with desired release profiles and methods of designing and making by Triastek Inc. (Shanghai, China) [166] and 3DP methods for developing compartmented pharmaceutical dosage forms providing a controlled release [167]. There are also patents from China featuring oral disintegrating tablets with amlodipine besylate prepared using 3DP [168] and orally disintegrating tablets with olanzapine and its fabrication using IJP [169].

### 8.3. Clinical Trials and Approved Drugs

**Figure 19:**
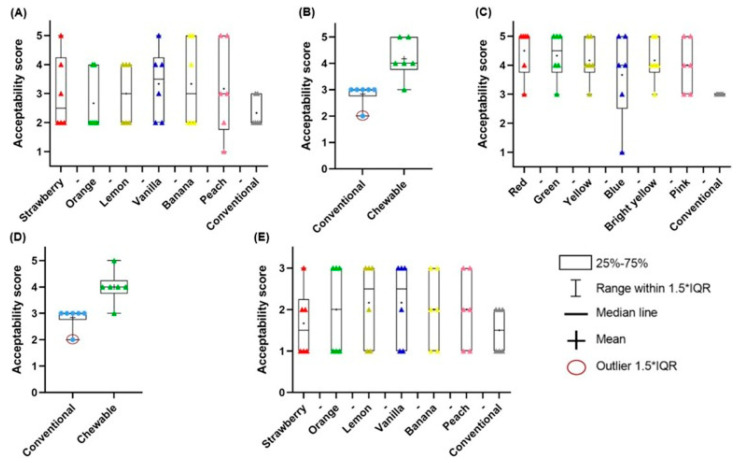
Representation of acceptability score of: (**A**) Flavour, (**B**) Shape, (**C**) Colour, (**D**) Texture and (**E**) Observations of facial expressions. Reprinted from [104] under a Creative Commons license.

The 3DP technology has been mainly explored by academic researchers. However, this technology attracted significant attention due to its application in preclinical research [170,171,172] and clinical trials [173]. The world’s first in-human clinical study involved chewable isoleucine printlets developed by FabRx Ltd.; the study was investigated at the Clinic Hospital at the University De Santiago de Compostela, Spain. The study concerned four different dosages (50, 100, 150, 200 mg) and six different flavours and colours; this personalized 3DP dosage form was prepared using SSE. Results showed desirable isoleucine blood levels and improved acceptability compared to the standard therapy [103]. Another clinical study was conducted on six children by Rodríguez-Pombo et al. [104] 3DP chewable medicines prepared in a hospital setting containing amino acids (citrulline, isoleucine, valine and isoleucine and valine combinations) were evaluated; the efficacy and acceptability were compared to conventional compounded medicines (Figure 19). After the therapy with 3DP drugs, the controlled amino acid levels were within target levels as well as the conventional medicines. Furthermore, according to children, the printlets were well accepted and, therefore can provide good adherence.

### 8.4. 4D Bioprinting

4D bioprinting is an innovative and progressive research approach that juxtaposes the combination of 3D printing along with the living cells and biomaterials, with the new component of time as the fourth dimension. In this way, it is possible to produce dynamic structures that exhibit the ability to change shape under the influence of external non-mechanical factors such as temperature, pH, humidity or electric or magnetic fields [174,175,176]. Using this advanced bioproduction method, scientists are able to design and construct a variety of models with applications not only in the biomedical field but also in pharmacy as modern drug delivery systems. The shape-morphing effect (SME) can be induced both by the individual properties of the biomaterials and by working external stimuli. There are specific types of SMEs, such as One-Way, Two-Way, or Multi-Way [177]. In addition to the need for the ability to change shapes and dimensions, it is important that the smart materials used are biocompatible, and should provide an environment that corresponds to physiological activity, especially in the case of artificial organ models, and more specifically, tissues. There are also various 4D bioprinting technologies, with inkjet bioprinting being one of the more widely used, due to its low cost and speed of printing. Worth noting is the question of the characteristics of the smart biomaterials used, which include shape memory polymers (SMPs) and shape morphing hydrogels (SMHs). The former can return to its fixed shape from a temporary form position under the influence of a response to external factors, while the latter has reversible swelling and shrinking properties. It is also worth pointing out the use of composite materials, which, unlike those previously mentioned, exhibit stronger mechanical and shape memory properties.

Currently, thanks to the 4D bioprinting method, it is possible to construct such models as those imitating skin, bone, blood vessels or muscle, among others. Such a solution allows not only to reproduce the physiological functions of these structures but also to monitor and control the release of administered drugs. Although 4D bioprinting is an extremely new technology proposing advanced solutions, it still requires many extensions and improvements in the performance of individual steps. Limitations are mainly the properties of biomaterials, which should have appropriate rheological characteristics, no toxicity to cells, and high printability and sensitivity to stimuli. Nevertheless, the development and optimization of the elements of this technology in the future will contribute to the improvement and better understanding of many medical cases, especially the individualization of therapy and the achievement of therapeutic purposes [174,175,176,178].

## 9. Conclusions

This noticeable growth in the research provides more and more insight into the area of 3DP, as well as pharmaceutical technology. Numerous paediatric medications may be developed using 3DP, by the pharmaceutical industry and researchers, paving the way for significant future innovations and addressing existing gaps in the availability of suitable medicinal products for children. Currently, the conventional DDS lacks acceptability, due to unpleasant taste or size. Furthermore, there is a risk of choking with the formulations, such as tablets and capsules. Consequently, the automated process of the layer-by-layer technique is capable of providing a solution, by producing customized, complex products, personalised for the patients’ needs. There is a noticeable increase in published papers on the 3DP of drugs, including the advancement in novel soft dosage forms for the paediatric population.

The emerging technique of 3DP of novel soft DDS has demonstrated high potential in overcoming challenges for paediatric patients. There is a visible predominance of gel formulations over non-gel-formulated. Consequently, there are new reports of gel-based chewable tablets and gummies for personalised use. The SSE technology implementation enables the creation of palatable dosage forms with customized doses, shapes, colours and textures through a quick and straightforward process. Moreover, it can utilize the same excipients as traditional chewable tablets, offering a significant advantage over conventional manufacturing techniques. In recent years, multiple 3DP advancements have been related to the non-gel-based novel DDS including the first attempts for the development of 3DP drug-loaded chocolates. Furthermore, the development of 3DP of chewing gum is observed, for the time being as a confectionery, but it may be a promising DDS, just like conventional MCG.

One of the promising areas that might benefit from the implementation of 3DP techniques is pharmaceutical compounding performed in community and hospital pharmacies. The potential of these methods has already been recognized and discussed [177], however, there are still a lot of practical and regulatory limitations preventing the application of 3D printers in pharmaceutical compounding. So far, among the most important obstacles, the lack of commercially available equipment and excipients suitable for the operation according to the pharmaceutical standards, no good quality control methods for the printed products and insufficient qualification of the medical and pharmaceutical staff should be mentioned. Moreover, these products would most probably require new regulations. The most recent studies show significant development in this area and prove that the difficulties described above can be overcome [179].

Additionally, the pharmaceutical industry started focusing on the development of 3DP for drug development, with the FDA’s approval of Spritam^®^ (Aprecia Pharmaceuticals, LLC, Langhorne, PA, USA) in 2015 as a significant milestone in 3DP application. The 3DP manufacturing process offers vital advantages, such as flexibility of dosage forms and tailored functionalities. Moreover, it provides more functionalities, including taste masking, modified release and enhanced bioavailability [180]. The ideal paediatric drug should offer dose flexibility, allowing for titration to accommodate a broad age range within the paediatric population using a single formulation [33]. Consequently, the industrial application of 3DP with the compatibility for high, low, ultra-low and fixed-dose enables a promising future and can significantly affect the development of drugs. There are many great promises in the 3DP. However, multiple technical and regulatory challenges still need to be addressed.

## Figures and Tables

**Table 1:** Comparison of different 3DP techniques.

Technique	Material Applied	Conditions	Potential Drawbacks	Advantages
FDM	thermoplastic polymer	high temperature	thermal decomposition of the drug	good equipment availability
SLA	photocurable resin	irradiation	resin toxicity	good equipment availability, good resolution
IJP	liquid ink	mild conditions depending on the material	nozzle clogging	good dose accuracy
SSE	gel/paste material	mild conditions depending on the material	low resolution, post-processing requirements	mild conditions, possibility to use biocompatible materials
DLP	photocurable resin	irradiation	resin toxicity	fast, cost-efficient, good resolution
e-3DP	viscoelastic ink and gel embedding phase	depending on the material	phases incompatibility, low resolution	interesting design, taste masking

**Table 2:** 3DP paediatric films.

Dosage Form	Active Ingredient	Printing Technique	Additives	Reference
hydrogel	L-ascorbic acid	SLA	PEDMA, triethanolamine, Riboflavin	[35]
gels	eucalypt extract	SSE	PEO, ascorbic acid, Eumulgin	[68]
buccal films	Miconazole	SSE	zein-PVP	[76]
oral films	Caffeine	SSE	SA, HPMC, SC	[47]
orodispersible mucoadhesive films	clobetasol propionate	DPE	HPMC, CS, HP-β-CD, PEO	[84]
buccal films with incorporated nanoparticles	Dolutegravir	SSE	PVAl, SA	[77]
ODF	paracetamol	hot melt ram extrusion	MDX, GLN, Span^®^ 80, GLY, TiO2	[83]
mucoadhesive buccal films	diclofenac sodium	FDM	PVAl, CS, EC, TEC, Xyl	[80]
FDF	paracetamol, ibuprofen	FDM	PVAl, PEO, ethylene, PEG, starch, sodium starch glycolate, croscarmellose, sodium lauryl sulfate	[91]

**Table 3:** 3D-Printed Gummies & Chewable Tablets.

Formulation	Active Compound	Printing Method	Printing Material	Reference
gummies	paracetamol and ibuprofen	e-3DP and SSE	water, glycerol, gelatine	[38]
gummies	ranitidine hydrochloride	SSE	corn starch	[94]
gummies	omeprazole	SSE	xanthan gum, corn starch	[95]
gummies	lamotrigine	SSE	gelatine, HPMC	[96]
gummies	simethicone	SSE	pectin	[93]
gummies	propranolol Hydrochloride	SSE	gelatin and γ-Aminobutyric acid	[97]
gummies	metformin	SSE and convective casting method	gelatine	[98]
chewable tablets	isoleucine	SSE	pectin, sucrose and maltodextrin	[103]
chewable tablets	citrulline, isoleucine, valine	SSE	pectin, sucrose, maltodextrin, water, maltitol	[104]
chewable tablets	hydrocortisone	SSE	pectin, sucrose, maltodextrin, water, maltitol	[105]
chewable tablets	amlodipine	SSE	CMC-Na, SSG, glycerine	[106]

**Table 4:** Novel 3D-printed non-gel formulations.

Formulation	Active Compound	Printing Method	Printing Material	Reference
chocolate	paracetamol, ibuprofen	extrusion-based 3DP	chocolate, corn syrup	[137]
chocolate	paracetamol, ibuprofen	extrusion-based 3DP	cacao, soy lecithin, glucose syrup	[138]
chocolate	paracetamol	SSE	chocolate	[139]
chocolate	VitD3	chocolate 3D printer	cacao, honey	[140]

## Data Availability

No new data were created or analysed in this study. Data sharing is not applicable to this article.

## Abbreviations

The following abbreviations are used in this manuscript:

3DP	Three-dimensional printing
AA	Ascorbic acid
AM	Additive manufacturing
API	Active pharmaceutical ingredient
CAD	Computer-aided design
CAF	Caffeine
CBS	Clobetasol propionate
CS	Chitosan
CMC-Na	Carboxymethyl cellulose sodium
DDS	Drug delivery system
DLP	Digital light processing
DNa	Diclofenac sodium
DOD	Droplets-on-demand
DOP	Drop-on-powder
e-3DP	Embedded 3D printing
EC	Ethyl cellulose
EE	Eucalypt extract
FDA	U.S. Food and Drug Administration
FDM	Fused deposition modeling
HP-β-CD	Hydroxypropyl-β-cyclodextrin
HPMC	Hydroxypropyl methylcellulose
ICH	International Congress on Harmonization
IJP	Inkjet 3DP
JH	Jerusalem sage honey
LH	Lavandula angustifolia honey
MSUD	Maple syrup urine disease
ODF	Orodispersible films
OLP	Oral lichen planus
PEG	Polyethylene glycol
PEO	Polyethylene oxide
PIB	Polyisobutylene
PLA	Polylactic acid
PVAc	Polyvinyl acetate
PVAl	Polyvinyl alcohol
PVP	Polyvinylpyrrolidone
R&D	Research and development
SA	Sodium alginate
SC	Sodium croscarmellose
SLA	Stereolithography
SLS	Selective laser sintering
SSE	Semisolid extrusion
TEC	Triethyl citrate
VitD3	Vitamin D_3_
VH	Vital honey
Xyl	Xylitol
SSG	Sodium starch glycolate
SBR	Styrene-butadiene
